# The analysis of National Health Accounts and financial communications network in Iran health insurance ecosystem

**DOI:** 10.1186/s12913-022-08921-3

**Published:** 2022-12-20

**Authors:** Rohaneh Rahimisadegh, Mohammad Hossein Mehrolhassani, Mohammad Jafari Sirizi, Somayeh Noori Hekmat

**Affiliations:** 1 Health Services Management Research Center, Institute for Futures Studies in Health, Kerman University of Medical Sciences, Kerman, Iran; 2 National Center for Health Insurance Research, Tehran, Iran; 3 Health Foresight and Innovation Research Center,Institute for Futures Studies in Health, Kerman University of Medical Sciences, Kerman, Iran

**Keywords:** Network Analysis, National Health Accounts, Cost, Ecosystem, And Health Insurance

## Abstract

**Background:**

One of the major reforms in the health system of any country is the financing reform. Network analysis as a practical method for investigating complex systems allows distinguishing prominent actors in the relation networks. Leading to the identification of the effective actors and key links between them, the analysis of financial networks helps policymakers to implement reformations by providing appropriate evidence. This study aimed to design and analyze the network of National Health Accounts (NHA) and the cost network in the Iran health insurance ecosystem.

**Methods:**

The present study is a network analysis study based on the data from NHA, and both cost and referral rates that was conducted in 2021. Data, which was for the years 2014 to 2018 and related to NHA, was collected from the Statistical Center of Iran, and cost data and referral rates, which were both related to Basic Insurance Organizations (BIOs), were collected from statistical yearbooks. To analyze the network and identify the key actors, macro indicators, such as network size and density, and micro indicators, such as centrality indicators and the combined importance index, were used.

**Results:**

In the financing of the health system in Iran, insurance organizations, as agents and sources of financing, do not have a very good position, so direct payments have become a key element in the network of NHA. Providing treatment-oriented services is quite prior. Regarded to health services, hospitals and outpatient services, such as pharmacies and physicians are the key elements of cost and referral rates respectively.

**Conclusion:**

Consisting of several organizations with different insurance policies and being supervised under different ministries, Iran's health financing system lacks a coherent structure. It is suggested to create a coherent insurance system by creating a single governance system and paying more attention to health-oriented instead of treatment-oriented services. The health insurance ecosystem has become a health-oriented system to reduce the direct payments as well as cost management.

## Introduction

According to the definition provided by the World Health Organization (WHO), the ultimate goal of health systems is to maintain and promote the health of individuals in societies, to meet their expectations in a justice-oriented manner, and protect them from harm and the financial burden of disease [1]. Financing is an essential component of health systems, but spending more resources in the health sector does not necessarily mean achieving better results and providing effective, efficient, and equitable health care for people in the health sector [2, 3]. Providing financial resources as one of the main tasks of a health system includes three main functions: revenue collection and financial resources, accumulation and management of revenues (risk accumulation), and allocation of resources to meet the health needs of individuals and society (service purchase) [4]. Achieving better and more results in the health sector and accordant with the goals of the health system requires the appropriate use of financing methods and policies [5].

In recent years, there has been a great deal of concern about financing the health sector in developing countries. To address this concern, new public and private insurance plans have been created to cover health costs [6]. Given that the financing of most health systems in the world, including Iran, is done through the insurance system, insurance organizations play a crucial role in achieving the goals and functions of the health system, especially health promotion, equitable financial participation, and financing [7]. Being an asset to support the health of individuals in communities, health insurance plays an intermediary role between the consumer and the health service provider and helps the people of society stay healthy and promote their health when they have an illness [1, 8, 9].

Health accounts play an important role in the discussion of financing health systems. Providing a basis for monitoring, and evaluating the capacity and sustainability of existing financing mechanisms, it can show how trends are costing [10]. NHA provides comprehensive and continuous information on the flow of financial resources in a country's health system and is one of the internationally accepted financing tools that are essential for the management of the health system [11]. Aiming to track, collect, measure, and estimate the financial flows of the health system by the four components of financial resources, financial factors, preventive and treatment providers, and health system functions, health accounts provide beneficial information for policymakers and decision-makers of a country throughout a particular time [12].

One of the most important reforms in a country's health system is the improvement and reformation of the financing system [13], which requires cooperation between different departments and organizations [14]. Given that any reformation requires information, NHA are considered a useful tool in performing health financing reforms, by which the following questions can be answered: How much is spent on health care? Who pays whom and for what? Who are the main actors in the health sector? Who are the main actors in the reformation process? [15].

Despite the importance of reforming the health financing system, the reformation process is difficult because many organizations and actors are involved in it [13, 16]. Therefore, the process of policy-making and implementation of policies in the field of reformations in mixed health systems such as in Iran requires governance institutions and multiple decisions due to the overlapping roles, diversity of responsibilities, and implementation methods [17]. To eliminate them, it is necessary to clarify and analyze the key actors, their roles and responsibilities, and their way of interaction in the network [18, 19].

Today, the network method has been developed as a very useful framework for studying complex systems because it allows the depiction of important actors in the network and other features of complex systems. Due to the professional mathematical foundations as well as graph and network theory and probabilities, the network method is unique in modeling complex systems [20]. In fact, by various effective elements existing in the health system systematical recognition, decision-makers and policymakers will have a better chance of implementing reformations [21]. Designing and analyzing the financial networks through the social network analysis method can identify the effective factors and key links and will help policymakers [22].

Despite the challenges in financing and the fact that NHA as a network reports the financial relationship between various factors (financial resources, financial agents, preventive and curative care providers, and health system functions), this study aimed to examine the health insurance ecosystem at the macro level of financing and specific at the level of payment insurance to provide a suitable background and evidence for reforms in the field of financing the health system by designing and analyzing the network of NHA and also investigating the position of insurance based on the data of statistical yearbooks of the health system.

### Study context (explanation about Iran's health and financing system)

Iran is an upper middle income country which population is estimated to be around 85,028,760 according to the World Bank in 2021 [23]. Healthcare services in Iran's health system are provided by government, private and charity sectors and at three levels [24]. Primary health care is provided in the form of a health care network, and secondary and tertiary level health care services are provided by hospitals, many of which are affiliated to the Ministry of Health and Medical Education (MoHME). The private sector is mainly active in providing secondary and tertiary level of healthcare services in urban areas [25]. In addition, inpatient healthcare services are most delivered by the government and public sector providers, and outpatient healthcare services are majority supplied by the private sector [24]. A significant part of the budget of the MoHME (about 22%) is spent on treatment [26]. According to the WHO's NHA data for 2019, Iran spent 6.7% of GDP on health. Also, according to this report, the main sources of financing health expenses include 49.5% of the financial contributions of the basic health insurance plans and the government budget, and 39.5% of out-of-pocket payments [27]. The population of Iran either includes people who are employed by the government and benefit from government health insurance plans with a relatively good budget, or includes people who have private health insurance, or people who do not have any insurance coverage and for receiving health services, they pay the costs directly from their own pockets [28].

According to the requirements of the fifth and sixth development plans of Iran to reduce out-of-pocket payments, increasing the government's share in financing public health expenses and appropriate allocation of public resources in the health sector is of great importance, and for this reason, providing fair health care services and accessible to the general public has been one of the concerns of the governments of the time and the realization of this goal has always faced serious challenges and obstacles [29]. The implementation of health system transformation plan in 2014 with the aim of reducing the costs of direct payment from the households' pockets, especially regarding treatment costs in the inpatient and hospital sectors, is considered as one of the important reforms in the Iran health financing system [26].

In Iran MoHME plays the main role both as a buyer and as a supplier as well as providing services to the majority of people. In addition, there are many other actors in the public and private sector as buyers, financiers and service providers, too. The payment system is considered a complex payment system with multiple financial flows to public and private providers from different financial sources and using different methods (for example, linear budget transfers, capital, fee for service and out-of-pocket payments) [18].

## Method

**Fig. 1:**
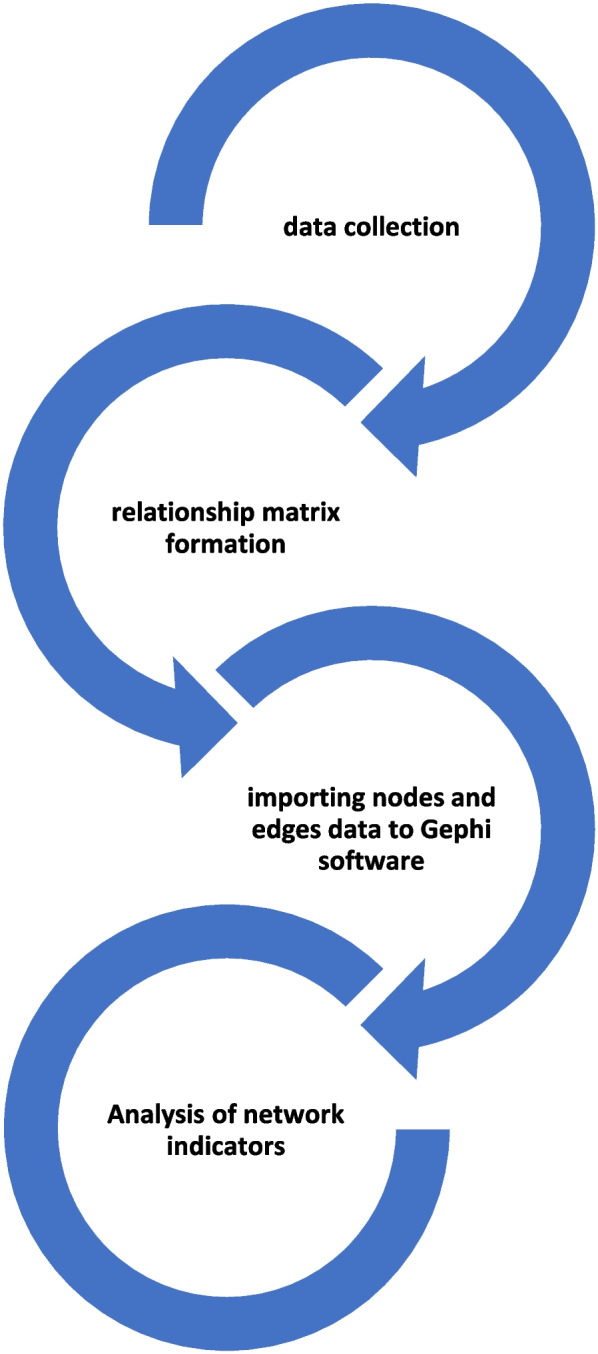
The study steps

The present study is a network analysis study based on the data of NHA, costs, and referral rates to investigate the financial relationship of health insurance ecosystem actors in 2021. The study steps are shown in Fig. 1.

In social network analysis, the network is considered as a set of nodes and edges between them. Nodes or actors are the units of analysis in networks and represent individuals, groups, communities, organizations or countries [30]. Edges defines the flow of material or non-material resources between actors [31] and resources may include social support, emotional support, time, information, expertise, money, business transactions, or performing joint activities [32]. There are two main ways to display networks. Relationship matrix and graph. In the first method, nodes and ties between them are shown in a matrix. In such a matrix, the nodes come in the first row and first column, and the numbers inside the matrix indicate the relationships between them. These numbers can be binary (zero and one) or weighted. Binary matrices only show whether there is a relationship between two nodes (network members). But in weighted matrices, weight can also be assigned to the relationship between two members. This weight can indicate the frequency of contact, the strength of communication, the duration of the relationship or a combination of these indicators [33]. In the second way of displaying the network, by using a graph, actors and their relationships such as the number of correspondences, financial or knowledge connections with other actors are visually displayed and analyzed [34].

In first step, data related to NHA were collected from the National Statistics Portal from 2014 to 2018. Cost and referral rates data, which were related to the main BIOs, were based on the statistical yearbooks uploaded on Iran Health Insurance Organization (IHIO) and Social Security Organization (SSO) websites.

In second step, to form a matrix of relations in the data related to NHA, the 4 main dimensions (financing agents, financial resources, health service providers, and functions) were added as network nodes to the row and column of the Excel file in a matrix of 52 by 52 (the nodes in NHA were 55, but after eliminating the total current health expenditure, the total and general health expenditure, which was the sum of the other options, 52 options remained as the nodes of the financial network). Then, the numbers in the tables of NHA were considered as the weight of the relationship between them and added to the matrix. The network depicted in Gephi software was a directional network showing the financial relationship between funding agents, financial resources, health care providers, and functions.

In order to form a matrix of relations of cost and referral rates data, the data related to the statistical yearbooks of insurance organizations was used. Due to the unavailability of the data related to Complementary Insurance Organizations (CIOs), these organizations were seen as nodes in the networks. But given that there was no data from them on the weight of their relationship with other nodes to be defined, these organizations are seen as isolated network nodes (without any connection to other nodes). In the cost data of the relations matrix between insurance organizations as service buyers and health service providers as service sellers, it was determined that the amounts paid by insurance organizations to each of the providers were defined in the matrix as the weight of their relationship. The number of network nodes was equal to 42, therefore a 42 by 42 matrix was defined in Excel, and cost numbers were entered as the weight of the connection between them.

In third step, the relations networks were designed separately for the year from 1993 to 1997 in Gephi software, but the results are shown on an annual basis since there was not much difference in the entirety of networks on an annual basis (although there was no significant difference in the results of every other year to show the approximate stability of the networks of NHA, cost, and referral rates period over 5 years).

In the final step, for identifying the most important, influential, and key actors in the network used indicators of social network analysis. Some indicators, such as network size (number of nodes and edges) and network density are related to the whole network and are called macro indicators. But some indicators, such as centrality indicators and clustering coefficient are at the level of network nodes and are called micro-indicators [35]. Table 1 shows the indicators used to analyze the NHA network in this study.

**Table 1:** Indicators used in the study to identify the most important actors

**Indicator type**	**Indicator**	**Definition**
Macro indicators	node	The basic unit and constituent of a network (actors) [36]
	Edges	Lines that connect two nodes, in which the links may have weight (importance, distance, etc.) in a network [36]
	density	This index is defined as the ratio of the number of all available links to all possible links [31]
Micro indicators	Degree centrality	The number of links that connect a particular node to other nodes. [31]
	Weighted degree centrality	When the links between the actors have weight, this index is obtained by multiplying the weight by the number of links that enter or exit a node [31]
	Closeness centrality	The sum of geodetic paths between a node and any other node in the network [31]
	Betweenness centrality	There are a number of other vertices that must pass through a particular node to reach their shortest [37]
	Page rank	The page rank index is calculated based on the relationship of each node in its weighted activity diagram and its measurement is calculated recursively [38]
	Clustering Coefficient	This indicator shows how the nodes are located next to their neighboring nodes [39]

Considering different aspects, the combined importance index was used to identify the most important nodes of NHA by considering several indicators of network analysis. The corresponding indicators for each, which leads to the identification of important network nodes, are shown in the below. It is shown that the importance index is the sum of the normalized value of each of these indicators.

Combined index of importance: normalized value of weighted degree + closeness centrality + betweenness centrality + page rank + clustering coefficient.

The following formula was used to normalize the values ​​of indices:

$$(\mathrm{Actual}\;\mathrm{value}\;\mathrm{index}\;-\;\mathrm{lowest}\;\mathrm{value}\;\mathrm{index})\;/\;(\mathrm{highest}\;\mathrm{index}\;\mathrm{value}-\mathrm{lowest}\;\mathrm{value}\;\mathrm{index})$$

It should be noted that in the cost network and referral rates, the number of isolated nodes (unrelated) was high, and the overall network density was very low (low network cohesion), so except for the Weighted Degree index, other indices were no longer calculated by the software. Therefore, only this index was used in the analysis of these networks.

## Results

To analysis the financial connections between the players of Iran's health insurance ecosystem, we used the method of network analysis in this study. In the results, first show the actors of Iran's health insurance ecosystem in the network of NHA, and then the most important actors of the network of insurers and service providers will be presented.

This study aimed to investigate the financial relations between the actors in the health insurance ecosystem. The relations of all actors in the context of NHA and the financial relations of insurance organizations with service providers were examined respectively.

As shown in Table 2, the most important actors in the NHA network, from 2014 to 2018, are outpatient service providers, hospitals, and health care providers, in which a total of three important actors in this network have the role of the service providers. Apart from households, other actors with the role of financial resources are not very important in the relation network. In the network of financial relations between the actors of the health insurance ecosystem, the position of health services concerning public health and prevention services is very important. In this relation network, the Social Security Organization (SSO) is considered a more important actor than the Health Services Organization (Health Insurance), so SSO is in the eighth place, and the Health Services Organization is in the eighteenth place in terms of importance index. The MOHME was ranked 23rd in the National Accounts Network because of its importance index and was very weak in 2016 of the 30% growth in playing a role as a mediator in establishing financial relations among other actors in 2014.

**Table 2:** The most important actors of the health insurance ecosystem in the NHA network

Id	Role category of actor	Actors	Importance			Weighted Degree			Closeness centrality			Betweenness centrality		
			2014	2016	2018	2014	2016	2018	2014	2016	2018	2014	2016	2018
20	health providers	Outpatient service providers	1/00	1/00	1/00	0/54	0/53	0/54	1/00	1/00	1/00	1/00	1/00	1/00
18	health providers	Hospitals	0/77	0/77	0/67	0/66	0/74	0/63	1/00	1/00	1/00	0/16	0/19	0/04
25	health providers	Institutions providing health related services	0/70	0/56	0/54	0/08	0/07	0/11	1/00	1/00	1/00	0/71	0/43	0/33
12	financial agencies	Direct payments	0/63	0/54	0/56	1/00	0/92	1/00	0/75	0/77	0/77	0/06	0/04	0/03
37	function	Medical services	0/63	0/54	0/59	0/96	1/00	0/96	0/00	0/00	0/00	0/00	0/00	0/00
23	health providers	Public Health and Insurance Department	0/62	0/55	0/51	0/03	0/04	0/05	1/00	1/00	1/00	0/30	0/38	0/27
21	health providers	Pharmacies and other retailers of medical supplies	0/60	0/49	0/51	0/28	0/31	0/31	1/00	1/00	1/00	0/00	0/01	0/03
9	financial agencies	SSO	0/59	0/51	0/45	0/35	0/43	0/27	0/78	0/73	0/73	0/41	0/31	0/27
19	health providers	Providers of nursing services and accommodation facilities	0/57	0/47	0/44	0/02	0/01	0/01	1/00	1/00	1/00	0/21	0/13	0/05
4	financial agencies	Other central government agencies	0/55	0/49	0/46	0/12	0/12	0/14	1/00	0/89	0/93	0/50	0/54	0/44
22	health providers	Developers and administrators of public health programs	0/54	0/40	0/41	0/04	0/01	0/00	1/00	1/00	1/00	0/04	0/02	0/01
24	health providers	Other activities	0/52	0/37	0/39	0/08	0/05	0/05	1/00	1/00	1/00	0/09	0/04	0/05
7	financial agencies	Municipality	0/50	0/37	0/41	0/00	0/01	0/01	0/75	0/70	0/83	0/48	0/36	0/35
16	financial agencies	Other organizations	0/49	0/28	0/27	0/11	0/08	0/07	0/70	0/71	0/70	0/45	0/15	0/12
26	health providers	Providers not categorized by function type	0/49	0/38	0/36	0/01	0/01	0/00	1/00	1/00	1/00	0/00	0/16	0/11
2	financial agencies	Universities of medical sciences under the MOHME	0/48	0/38	0/39	0/48	0/45	0/44	0/75	0/79	0/80	0/04	0/04	0/04
13	financial agencies	Non-profit organizations serving households	0/49	0/46	0/44	0/00	0/00	0/00	0/65	0/64	0/65	0/49	0/49	0/41
8	financial agencies	Health Services Organization	0/48	0/38	0/35	0/25	0/34	0/26	0/81	0/80	0/83	0/22	0/12	0/12
3	financial agencies	Armed Forces	0/41	0/29	0/24	0/06	0/06	0/06	0/81	0/75	0/70	0/20	0/17	0/08
14	financial agencies	Banks	0/40	0/24	0/23	0/03	0/03	0/03	0/81	0/79	0/76	0/20	0/08	0/03
11	financial agencies	Private CIOs	0/40	0/28	0/32	0/06	0/08	0/16	0/88	0/82	0/85	0/09	0/08	0/11
10	financial agencies	Government CIOs	0/40	0/26	0/23	0/08	0/07	0/03	0/80	0/75	0/71	0/08	0/05	0/03
1	financial agencies	MOHME	0/36	0/37	0/34	0/03	0/04	0/03	0/79	0/83	0/83	0/06	**0/33**	0/27
42	function	Public health and prevention services	0/31	0/14	0/14	0/12	0/08	0/07	0/00	0/00	0/00	0/00	0/00	0/00
32	financial sources	Households	0/29	0/19	0/20	0/40	0/38	0/41	0/59	0/58	0/58	0/00	0/00	0/00

**Fig. 2:**
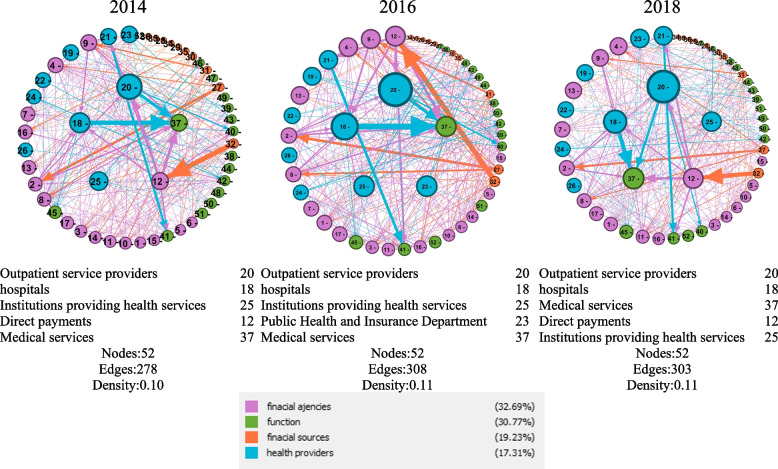
The network of elements of the health insurance ecosystem in the NHA context

As can be seen in Fig. 2, direct payments in the NHA network in 2014 and 2018 had a higher rank in terms of the importance index, which decreased in 2016 and was removed from the list of 5 important actors in the network.

Households, as one of the actors in the NHA network shown in the networks with node 32, have a strong relationship with direct payments shown in Fig. 2 with a thick orange line from node 32. Households are connected to node 12 (direct payments). Another strong link in the NHA network is the link between node 18 (hospitals) and node 37 (health services).

Although the group of health service providers allocate for only 17% of the total network of NHA, they have the most important actors in the network, which is the opposite of the group of functions.

Another network examined in this study as a small part of the NHA network was the financial relation network between insurance organizations and service providers in the form of service delivery and purchase services, whose results will be presented later.

As seen in Table 3, the most important actor in the network in terms of cost network from 2014 to 2018 is IHIO, followed by hospitals and limited surgery centers in the relation network of actors of NHA. Village-level health centers providing health and prevention services are less important than hospitals providing health care.

**Table 3:** The most important actors of the health insurance ecosystem in the cost network

Id	**Actors**	**Weighted Degree**^**a**^		
		**2014**	**2016**	**2018**
12	**IHIO**	103,226,078	148,175,449	142,412,786
1	**Hospitals and limited surgery centers**	92,423,700	136,052,900	130,314,600
13	**SSO**	77,381,064	128,463,267	137,230,563
5	**Pharmacies**	22,394,950	37,499,400	42,089,200
11	**Health Centers**	17,355,700	23,659,700	22,911,700
3	**SPs**	15,669,790	26,064,290	26,921,640
2	**GPs**	14,104,510	20,552,980	21,531,130
7	**Radiology centers**	4,767,880	9,720,970	9,410,370
6	**Lab centers**	4,373,950	7,774,520	7,611,760
9	**Dialysis centers**	3,380,086	4,743,417	6,766,031
4	**Dentists**	3,074,741	4,920,481	5,256,980
8	**Rehabilitation centers**	2,328,426	4,552,822	5,475,619
10	**Others centers**	733,409	1,097,236	1,354,319

^a^Costs have been reported in millions of Rials

**Fig. 3:**
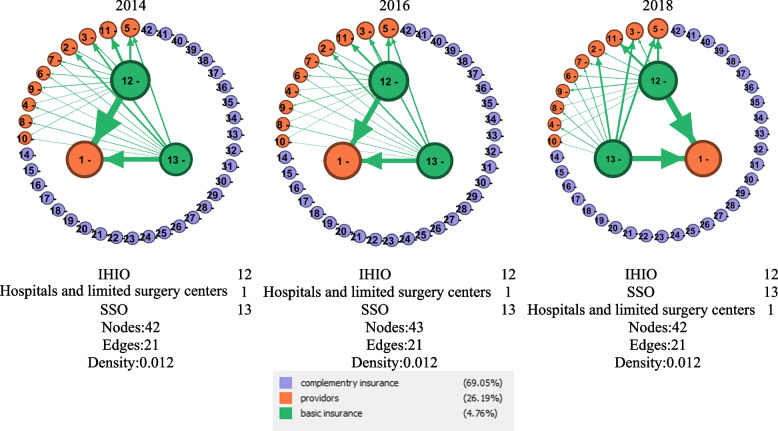
Cost network between insurance organizations and health service providers in the health insurance ecosystem

As shown in Fig. 3, the three main actors in the cost network between insurance organizations and health service providers in the health insurance ecosystem from 2014 to 2018 have remained unchanged, and the strongest network relations between them have been established. A large share of this network (69%) is to the supplementary insurance organizations in terms of the number of actors, which due to lack of access to their cost data (concerning the competition between CIOs in having more customer acquisition and share of the market), are seen as the isolated network nodes.

In the referral rates network, the most important actors after insurance organizations are pharmacies, specialist physicians (SPs), and general physicians (GPs). Comparing the number of referrals of insurers shows that the number of referrals of SSO is higher than that of IHIO. It also increased from 2014 to 2018, while the number of referrals to IHIO in 2016 increased from 2018 and decreased again in 2018 (Table 4).

**Table 4:** The most important actors of the health insurance ecosystem in the referral rates network according to the Weighted Degree index

Id	**Actors**	Weighted Degree		
		2014	2016	2018
13	SSO	258,736,373	306,080,878	323,688,134
12	IHIO	146,452,507	199,246,093	148,301,315
5	Pharmacies	114,906,573	183,300,267	166,644,780
3	SPS	99,141,152	115,050,784	108,527,735
2	GPs	98,718,114	101,594,763	92,118,675
6	Lab centers	37,303,404	40,935,633	39,113,422
7	Radiology centers	24,821,944	29,777,485	26,164,508
9	Dialysis centers	8,839,299	10,969,787	14,452,065
1	Hospitals and limited surgery centers	7,978,609	10,058,477	9,297,469
4	Dentists	7,399,975	6,300,451	5,012,652
10	Others centers	5,268,550	6,253,425	9,434,655
8	Rehabilitation centers	811,260	1,085,900	1,223,489

**Fig. 4:**
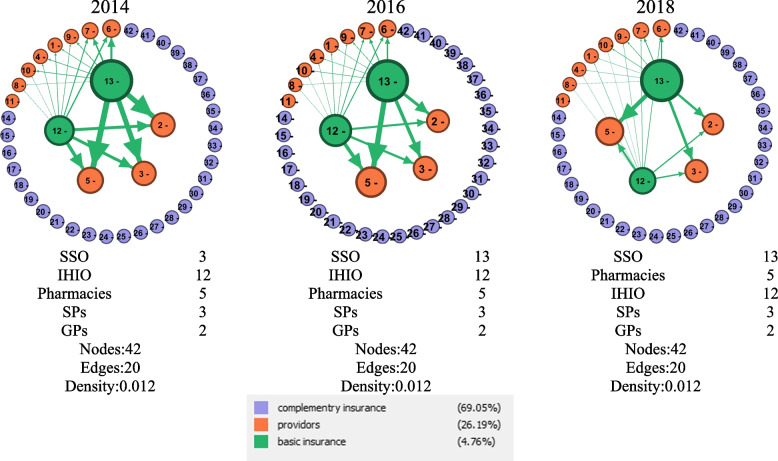
The network of referral rates of insurance organizations and health service providers in the health insurance ecosystem

The thickness of the relation lines between the network nodes shown in Fig. 4, indicates the intensity and weight of the relation between network actors, which means that the number of times the SSO insured referred to service providers has more weight than the IHIO insured.

## Discussion

The function of financing as one of the important functions of the health system includes many actors and elements that the key elements and how they relate to each other in the relation network should be examined before making any reforms in this field. This trend is recently used in many countries, too. In this study, the fragmentation of the health insurance ecosystem in the Iranian health financing system at both macro and micro levels was shown by analyzing social networks, the networks of NHA, cost networks, and referral rates, which provides evidence and information for policy-makers to improve health system financing reforms. In the following, the results of the study will be discussed at two levels: macro (NHA network) and micro (network of relations between insurance and providers).

### NHA network

In the NHA network, the direct payments by households were identified as the most important source of funding. The outpatient service providers and hospitals were also identified as the most important providers of health services. While evidence suggests that direct payments are not only the most unfair but also the most inefficient method of financing, direct payments are usually the largest or second largest source of funding for the health system in developing countries [1, 40]. Having a fairer and more efficient financing system in the health system requires the development of effective financing strategies that will be done in a more organized way, such as prepayment mechanisms and risk accumulation using insurance plans instead of direct payments at the time of service [41, 42]. In NHA, households usually act as sources of financing in addition to their role as financing agents. But when households pay for health expenses accumulatively in advance by social health or private insurance plans, households act only as sources of financing, which reduces the direct payments [40].

In the present study, the results showed that in the NHA network, direct payments were one of the key elements of the network in 2014. According to the direct payments reduction which was one of the goals of the health system transformation plan after its execution in 2016, their importance was reduced and got removed from the 5 key elements of the network [41, 43, 44]. The results of the studies indicate that according to the total costs of the health sector, the implementation of the health system transformation plan not only did not reduce the direct payment, but did increase it in the private sector, especially in hospitals and outpatient services after the tariff increases in 2015 [45]. According to the results of the present study in 2018, the direct payments were added to the 5 key elements of the network again.

In the NHA network, the component of health service providers was more important than the other three components (financing agents, financial resources, and operations) in the network from 2014 to 2018. For example, outpatient service providers and hospitals are two important elements of the network in these years. In the cost network between insurance and health service providers, hospitals were also recognized as the key elements of the network, which seems to be more important and prior than health services and disease prevention in Iran. A study of NHA from 2002 to 2011 in Iran has shown that 80% of health expenditures were allocated to health services (63%) and medicine (17%) [46].

### Cost and referral rates network

The comparison of cost and client relations networks shows that even a slight change in the important actors of the network did not occur between 2014 to 2018. Health insurance and SSO, as the two main BIOs in Iran, are considered two important basic actors in this period. In the cost network, hospitals and limited surgery  centers, and in the network of referral rates, pharmacies, GPs, and SPs are the key ones. It is thought to have a more important position in the interactions between insurance organizations and providers in inpatient and hospital services concerning costs and outpatient services concerning referral rates.

Health insurance coverage in Iran is provided by three main sectors including BIOs, institutional or institutional health insurance funds, and CIOs. BIOs include organizations, such as IHIO (under the supervision of the MoHME), SSO (under the supervision of the Ministry of Welfare and Cooperation), and AFMSO (the Armed Forces Medical Services Organization under the Ministry of Defense). According to the Universal Health Insurance Act, they are required to provide basic health care services. Institutional health insurance funds several organizations, such as the Oil Industry Health Organization, banks, etc., which specifically provide services for their employees, and some organizations, such as CIOs (under the Ministry of Economy and Finance), Alborz, Mellat, Pasargad, Atieh Sazan Hafez, Dana, etc., which provide services that do not provide BIOs [47]. As can be seen, the structure of the health insurance ecosystem in Iran is fragmented and has created many challenges for the country's financing system [48].

Another challenge of the Iranian health insurance ecosystem is its treatment-oriented services focusing more on the provision of medical services than on the health and preventive services, which is also confirmed by the results of our study [49]. Up to 2018 outpatient services, which include pharmacies, specialists, and subspecialty physicians, are the most important and are considered one of the 5 key elements of the network. And inpatient services, such as hospitals and limited surgery centers are considered important in the cost network.

In the cost network, IHIO was recognized as one of the most important elements in all the three networks in 2014, 2016, and 2018. The insured of IHIO of Iran use private hospitals or university hospitals under the supervision of MOHME (Ministry of Health and Medical Education) to use inpatient services, which increases the possibility of increasing costs and direct payments. However, people covered by SSO do not need to pay for treatments by being admitted to civilian social security hospitals (direct treatment). Therefore, costs and direct payments in this organization are reduced compared to IHIO [50].

Due to the different policy structures of insurance organizations in Iran in presenting and reporting their financial data, there was no access to the cost data of CIOs and AFMSO. Therefore, in the cost network between insurance organizations and service providers, CIOs were displayed as isolated nodes. The Weighted Degree index was used to determine the important actor in these networks, and AFMSO was only displayed on the NHA network.

## Conclusion

The financing system of the Iranian health system lacks a coherent and coordinated structure, and the role of insurance as a financing factor is weak. Accordingly, the structure of the current insurance ecosystem is incoherent and fragmented, consisting of various organizations with different insurance policies under the supervision of several ministries. Since the role of direct payments in the network of NHA was very colorful, it is thought that insurance organizations have a terrific position both as financing agents and sources in the financing system of the Iranian health system. Do not forget that this issue makes direct payments a key element in the network of NHA important and highlights their line of relations with households as both financing agents and sources in this network. Also, in the Iranian health insurance ecosystem, the provision of treatment-based services has a higher status and priority than health-based services. Thereby, hospitals in terms of cost, and outpatient services, such as pharmacies and physicians in terms of referral rates are the key actors.

It is suggested that a coherent insurance system be created through a single government system due to the fragmentation of the country's health insurance system. It is recommended that the health insurance ecosystem become health-oriented rather than treatment-oriented by paying more attention to health-oriented services. So, not only does it manage costs, but also can it reduce the number of direct payments.

It is also suggested that in the future studies, the analysis of the health insurance ecosystem network and the financing of the health system be examined by using individual data at a micro-level to analyze the relation network between physicians, pharmacies, hospitals.

## Data Availability

The data analyzed is available from the corresponding author on reasonable request.

## Abbreviations

NHA: National Health Accounts
IHIO: Iran Health Insurance Organization
SSO: Social Security Organization
MOHME: Ministry of Health and Medical Education
BIOs: Basic Insurance Organizations
CIOs: Complementary Insurance Organizations
AFMSO: Armed Forces Medical Services Organization
GPs: General Physicians
SPs: Specialist Physicians
WHO: World Health Organization
